# LncRNA XIST from the bone marrow mesenchymal stem cell derived exosome promotes osteosarcoma growth and metastasis through miR-655/ACLY signal

**DOI:** 10.1186/s12935-022-02746-0

**Published:** 2022-10-29

**Authors:** Guanghui Zhu, Yu Xia, Ziyue Zhao, Aoyu Li, Hui Li, Tao Xiao

**Affiliations:** 1 Department of Orthopaedics, The Second Xiangya Hospital, Central South University, 139 Renmin Road, Changsha, 410011 Hunan People’s Republic of China; 2 Department of Pediatric Orthopedics, Hunan Children’s Hospital, Changsha, Hunan People’s Republic of China; 3 Orthopedic Biomedical Materials Engineering Laboratory of Hunan Province, Changsha, Hunan People’s Republic of China

**Keywords:** Bone marrow mesenchymal stem cells, Osteosarcoma, Exosome, LncRNA XIST, miR-655

## Abstract

**Background:**

Long non-coding RNA X-inactive specific transcript (XIST) regulates the progression of a variety of tumors, including osteosarcoma. Bone marrow mesenchymal stem cells (BMSCs) can be recruited into osteosarcoma tissue and affect the progression by secreting exosomes. However, whether BMSCs derived exosomes transmit XIST to regulate the growth and metastasis of osteosarcoma and the related mechanism are still unclear.

**Method:**

In this study, BMSCs derived exosomes were used to treat human osteosarcoma cells MG63 and 143B, and the level of XIST in BMSCs was intervened by siRNA. CCK-8, EdU, transwell assays were used to analyze the changes of cell proliferation, migration and invasion. Bioinformatics analysis, RNA pulldown and dual-luciferase reporter gene assays validated the targeted relationship of XIST with miR-655 and the interaction between miR-655 and ACLY 3’-UTR. 143B/LUC cell line was used to establish an animal model of in situ osteosarcoma to verify the found effects of XIST on osteosarcoma. Oil Red O staining, Western blot and so on were used to detect the changes of lipid deposition and protein expression.

**Results:**

It was found that BMSCs derived exosomes promoted the proliferation, migration and invasion of osteosarcoma cells, and the down-regulation of XIST inhibited this effect. miR-655 mediated the role of BMSCs derived exosomal XIST in promoting the progression of osteosarcoma and down-regulation of miR-655 could reverse the effects of inhibiting XIST on the proliferation, migration and invasion of osteosarcoma cells. Meanwhile, animal level results confirmed that BMSCs derived exosomal XIST could promote osteosarcoma growth and lung metastasis by combining with miR-655. In-depth mechanism study showed that BMSCs derived exosomal XIST combined with miR-655 to increase the protein level of ACLY, which led to lipid deposition and activate β-catenin signal to promote the proliferation, migration and invasion of osteosarcoma cells.

**Conclusion:**

This study showed that BMSCs derived exosomal XIST could enter osteosarcoma cells, bind and down-regulates the level of miR-655, resulting in an increase in the level of ACLY, thus increasing the lipid deposition and the activity of β-catenin signal to promote the growth and metastasis of osteosarcoma.

**Supplementary Information:**

The online version contains supplementary material available at 10.1186/s12935-022-02746-0.

## Introduction

Osteosarcoma is a common malignant bone tumor in children, adults and young adults [1]. Surgical resection and neoadjuvant chemotherapy are the main treatment methods for newly diagnosed osteosarcoma patients [2]. Although the 5-year survival rate is 60–70%, many patients still die of the rapid progress and strong invasiveness of the disease [3, 4]. New therapeutic methods are urgent to be developed, and its pathological mechanism is the basis for the development of new therapeutic strategies.

Bone marrow mesenchymal stem cells (BMSCs), derived from bone marrow stroma, as a kind of pluripotent stem cells, can differentiate into a variety of terminal effector cells, such as osteoblasts and adipocytes [5]. Studies have shown that BMSCs can target and migrate to tumor sites. As one of the components of tumor microenvironment, BMSCs regulate multiple processes of tumor progression [6]. In osteosarcoma, BMSCs can be recruited to tumor sites, which plays a critical role in osteosarcoma malignant [7]. Therefore, it is particularly necessary to deeply unveil the mechanism of BMSCs promoting the progression of osteosarcoma.

Exosomes are extracellular vesicles with a diameter of 40-100 nm secreted by cells and mediate cell–cell interaction [8]. Importantly, current studies have confirmed that exosomes from BMSCs promote the progression of osteosarcoma [9, 10]. The function of exosomes is related to their cell surface molecules and cellular contents. Long non coding RNA (LncRNA) is one of the contents [11]. Exosomal lncRNA participates in the regulation of the process of a variety of diseases by mediating cell–cell interactions, including tumors [12]. For example, macrophage derived exosomes can enter osteosarcoma cells by transferring LIFR-AS1, and combine with miR-29a to promote the expression of NFIA, promote cell proliferation, invasion and inhibit apoptosis [13]. The key is that BMSCs exosomes can enter osteosarcoma cells with transferring lncRNA PVT1. On one hand, PVT1 can combine with miR-183 to promote ERG expression, on the other hand, PVT1 can directly combine with ERG to inhibit its ubiquitination degradation, and finally enhance the growth and metastasis of osteosarcoma [14]. This study preliminarily showed that lncRNA mediates the role of BMSCs derived exosomes in regulating the progression of osteosarcoma. However, little details is known about this, so more and more in-depth studies are needed to unveil other lncRNAs that mediate exosomes from BMSCs to regulate the progression of osteosarcoma, and their mechanisms.

Long non coding RNA X-inactive specific transcription (XIST) is one of the earliest lncRNAs ever found to play a key role in X chromosome inactivation, which affects the progression of a variety of tumors [15]. In osteosarcoma, most studies have shown that XIST plays a role in promoting cancer. For example, in osteosarcoma, the expression of XIST is up-regulated, which is closely related to tumor size, clinical stage and distant metastasis [16, 17]. In vitro and in vivo studies have found that XIST promotes the progression of osteosarcoma, and its mechanisms include promoting Rab16 expression by binding miR-758, promoting SNAI1 expression by binding miR-153, and inhibiting NF-κB/PUMA signal can further antagonize apoptosis and mediate the regulation of AGO2 expression by HuR [18–21]. Importantly, XIST can exist in exosomes, and the level of XIST in serum exosomes of patients with recurrent triple negative breast cancer (TNBC) is higher than that of patients without recurrence [22]. However, it is unclear whether BMSCs derived exosomes affect osteosarcoma progression through XIST.

In this study, we intended to isolate BMSCs derived exosomes, treat osteosarcoma cells with these exosomes, observe the changes of XIST level in osteosarcoma cells, analyze the effects of BMSCs derived exosomes on osteosarcoma growth and metastasis through XIST, and then study the mechanism of BMSCs derived exosome XIST regulating osteosarcoma progression, unveil the downstream molecules and signal pathways.

## Materials and methods

### Cell culture

Human BMSCs(Procell, CP-H166) were cultured in DMEM/F12 (Procell, PM150310) plus 10% FBS (Procell, 164210-500). The cell lines MG63 and 143B were obtained from American type culture collection (Manassas, VA), the cell line 143B/LUC was purchased from Ming Zhou Bio., all cell lines were cultured in MEM (Procell, PM150410) plus 10% FBS.

### Characterization of BMSCs

BMSCs were characterized by detecting cell surface antigen using flow cytometry (BD, C6). Briefly, BMSCs at passage 3 reaching 90% confluence were washed twice with phosphate buffered saline (PBS), digested with 0.25% trypsin, collected after digestion, centrifuged at 1500 rpm for 5 min to remove the supernatant, and then washed twice with PBS and centrifuged again. After cell counting, the cell concentration was adjusted to 1 × 10^5^ cells/mL. Then the primary antibodies (CD29, Invitrogen, 11-0299-42; CD45, Invitrogen, 11-0459-42; CD90, Invitrogen, 11-0909-42) were added and the cells were incubated in the dark for 30 min at 4 °C. After that, the cells were centrifuged at 1500 rpm for 5 min to remove the supernatant, and PBS was added to resuspend for cleaning and the cells were centrifuged again to remove the supernatant. 200 μL PBS was added to resuspend the cells and the samples were detected by flow cytometry to analyze the proportion of CD29, CD45 and CD90 positive cells.

### Osteogenic differentiation of BMSCs

BMSCs at passage 4 were seeded in six-well plates at the concentration of 3 × 10^4^ cells/mL and treated with osteogenic induction medium (Procell, PD-007) for 14 days. The cells were collected and tested according to the instructions of the alkaline phosphatase (ALP) kit (Changchun Huili, C003-b). The absorbance (OD) at 450 nm was measured with a microplate reader (MD, Flexstation 3) to evaluate the ALP level. The osteogenic induction medium treated the cells for 21 days. The cells were fixed with 4% paraformaldehyde (Sinopharm group, 80096618), stained with 1% alizarin red (Sigma-Aldrich, St. Louis, Mo) for 5 min to show mineralized nodules. The staining results were observed under an inverted microscope and photographed.

### Oil Red O staining

BMSCs at passage 5 were seeded 6-well-plates at the concentration of 2 × 10^4^ cells/mL and maintained in adipogenic differentiation basic medium A (Procell, PD-019) for 3 days, followed by 1 day in adipogenic differentiation basic medium B (Procell, PD-019). Both steps were repeated up to 14 days (indicated as the 3rd cycle). Fixed cells with 4% paraformaldehyde and stained with Oil Red O (Servicebio, G1015) for 30 min. The staining results were observed under an inverted microscope and photographed. For tumor tissue samples, the tissue was fixed with 4% paraformaldehyde for 24 h, embedded in paraffin, sliced to 4 μm sections, stained with Oil Red O for 30 min, observed under an inverted microscope and photographed.

### Isolation and characterization of exosomes

BMSCs at passage 3–5 were cultured in DMEM/F12 with 10% FBS (SBI, EXO-FBS-50A-1) without exosomes. After 48 h, the supernatant of culture medium was collected, centrifuged at 300 g at 4 °C for 10 min to remove possible cell components, then centrifuged again at 2000 g for 10 min to remove cell debris, and then centrifuged under 10000 g at 4 °C for 30 min to remove large membrane vesicles. The obtained supernatant was then centrifuged at 120000 g for 70 min at 4 °C. After removing the supernatant, the bottom sediment was resuspended in PBS, and then the sample was ultracentrifuged at 140000 g for 90 min at 4 °C. The bottom sediment was collected as the exosome sample. The isolated BMSCs exosomes were incubated overnight with 2.5% glutaraldehyde solution at 4 °C, then fixed with 1% osmium tetroxide for 1.5 h, stained with 1% uranyl acetate (pH4.0), observed under transmission electron microscope (JEOL, JEM-2100 plus) for morphology and size, and photographed at 200 kV. The levels of exosome specific markers CD9, CD63 and CD81 were detected by Western blot, and the endoplasmic reticulum marker Calnexin was used as the negative control.

### Exosome uptake

PKH26 Red Fluorescent Cell Linker Minin Kit (Sigma, MINI26) was used to exosomes label: 500 μL diluent C solution was added to 200 μL exosome suspension, a 1.5 mL Eppendorf (EP) tube was prepared, 4 μL PKH26 dye and 500 μL diluent C solution was added to it. The above two liquids were mixed well in the dark. After 5 min, 1 mL of 10% bovine serum albumin was added to the mixture to stop dyeing. A 0.22 μm cell membrane was put on a small ultracentrifuge tube, and 6 mL PBS was added to it, and the sample was centrifuged at 100000 g for 90 min, and the sediment was collected and resuspended with PBS, and the sample solution was stored. The steps of the uptake of exosomes by osteosarcoma cells were as follows: MG63 and 143B cells were allowed to proliferate to 70% confluence in 12-well-plates, and the medium was replaced with the fresh culture medium supplemented with PKH26 labeled exosomes. After incubated for 12 h, the cells were washed twice with PBS, then fixed with 4% paraformaldehyde for 20 min, the nuclei were stained with 4’,6-diamidino-2-phenylindole (DAPI). Finally, the cells were observed and photographed under the laser confocal fluorescence microscope (Olympus,BX53).

### RNA isolation and qRT-PCR

Total RNA was extracted from cell or tissue samples by Trizol (Invitrogen™). Exosomal RNA was extracted with a kit (Norgen BioTek, NGB-58000). The first strand cDNA synthesis reaction system was prepared in RNase free PCR tube, and theEasyScript first strand cDNA synthesis Supermix k it is used to reverse transcribe RNA into cDNA. SYBR Green qPCR Supermix and Applied Biosystems® 7500 sequence detection system was used to detect the RNA level. GAPDH was used as the internal reference of XIST, and U6 was used as the internal reference of miRNA. PCR primers were purchased from genscript Biotechnology Co., Ltd. The primer sequences were: XIST forward 5’-ACGCTGCATGTGTCCTTAG-3’ and reverse 5’-GAGCCTCTTATAGCTGTTTG-3’; GAPDH forward 5’-TCAAGAAGGTGGTGAAGCAGG-3’ and reverse 5’-TCAAAGGTGGAGGAGTGGGT-3’; miR-655 forward 5’-TGCGCATAATACATGGTTAACC-3’; miR-374c forward 5’-TGCGCATAATACAACCTGCTAA-3’; miR-5590 forward 5’-TGCGCAATAAAGTTCATGTAT-3’; miR-129-1 forward 5’-TGCGCAAGCCCTTACCCCAAAA-3’; miR-129-2 forward 5’-TGCGCAAGCCCTTACCCCAAA-3’; reverse 5’-CCAGTGCAGGGTCCGAGGTATT-3’;U6 forward 5’-CGCTTCGGCAGCACATATAC-3’ and reverse 5’-AAATATGGAACGCTTCACGA-3’. Relative expression was calculated using the 2^−△△CT^ method.

### Western blot

The total proteins of exosomes, cells and tissues were extracted with RIPA lysate (Beyotime, P0027), and the cytoplasm and nuclear proteins were extracted with NE-PER™ nuclear and cytoplasmic Extraction Reagent (Thermo Scientific™, 78833). Protein concentration was measured with the BCA protein assay kit (Beyotime, P0012S). Protein was separated by 10% sodium dodecylsulfate-polyacrylamide gel electrophoresis (SDS-PAGE). Then, the protein was transferred to PVDF membrane (Immobilon-P, Millipore, Billerica, MA, United States) with the electrical transferring apparatus. The PVDF membrane was incubated with primary antibodies diluted with blocking solution overnight at 4℃ respectively (GAPDH, Abcam, ab8245; Lamin B1, Abcam, ab16048; β-catenin, Abcam, ab32572; CD9, Abcam, ab263019; CD63, Abcam, ab134045; CD81, Abcam, ab79559; calnexin, CST, 2679; ARPP19, Affinity, DF9325; TOB1, ProteintechGroup, Inc, 14915-1-AP; ACLY, Abcam, ab40793. the dilution ratio was 1:1000), and the corresponding HRP labeled secondary antibody (HRP labeled Sheep anti-mouse secondary antibody, Wuhan Boster Biological Technology., Ltd., BA1051; HRP labeled Sheep anti-rabbit secondary antibody, Wuhan Boster Biological Technology., Ltd., BA1054; the dilution ratio was 1:5000) was diluted with the blocking solution and the PVDF membrane was incubated at room temperature. The enhancer in ECL reagent was mixed with stable peroxidase solution at the ratio of 1:1, and the mixture was dropped onto the PVDF membrane. After the X-ray film was pressed, the film was successively put into the developing solution for development, fixer for fixation, and the film is developed. Western blot results were analyzed using Image J software (National Institutes of Health, USA).

### Fluorescence in situ hybridization

Cy3-labeled oligonucleotide probes for XSIT was applied for RNA FISH, the probes were designed and synthesized by Genepharma (Shanghai, China). Cells were seeded in a glass-bottom dish. Then the cells were incubated with prehybridization solution at 37 °C for 30 min and the probes were added to dish and the hybridization was performed overnight. Then the cells were washed with buffer I (4 × SSC, 0.1% Tween-20) for 3 times, wash with buffer II (2 × SSC) once, and wash with buffer III (1 × SSC) once. After being washed with phosphate-buffered saline, the cells were incubated with DAPI to stain cell nuclear. The images were acquired on confocal microscope.

### Cell transfection

The transfection was performed after the confluence of cells reached 70%. XIST specific interfering siRNA, miR-655 agomir and antagomir were all purchased from Genepharma (Shanghai, China), and the vectors were constructed in this laboratory. Lipofectamine™ 3000 (Invitrogen™) was used for transfection, and the follow-up experiment was carried out 72 h later. XIST siRNA sequence: siNC: 5'-UUCUCCGAACGUGUCACGUdTdT-3'; siXIST#1: 5'-GUAUCCUAUUUGCACGCUAdTdT-3'; siXIST#2: 5'-GCCCUUCUCUUCGAACUGUdTdT-3'; siXIST#3: 5'-GUAUCCUAUUUGCACGCUAdTdT-3'. miR-655, sense 5’-AUAAUACAUGGUUAACCUCUUU-3’ and antisense 5’-AGAGGUUAACCAUGUAUUAUUU-3’; NC/Anti-NC, sense 5’-UUCUCCGAACGUGUCACGUTT-3’ and antisense 5’-ACGUGACACGUUCGGAGAATT-3’; Anti-miR-655, 5’-AAAGAGGUUAACCAUGUAUUAU-3’.

### CCK-8

Cell viability was accessed by using a CCK-8 (MCE, HY-K0301). After the cells were digested with trypsin, the cell concentration was adjusted to 5 × 10^4^ cells/mL. The cell was inoculated in 96-well-plates at 100 μL per well. The cell suspension and blank group were set at the same time. 10 μL CCK-8 reagent was added to each well and the plate was incubated for 2 h in the incubator. Finally, the absorbance at 450 nm of each well was measured with the microplate reader.

### EdU

The cells were inoculated in 96-well-plates (5000 cells per well) and incubated for 24 h. The cells were then incubated in 10 μM EdU reagent for 4 h, fixed with 4% paraformaldehyde, permeated by 0.5% Triton X-100, and stained with 1 × Apolloreagent for 30 min. Nuclei were stained with 1 × DAPI, and the cells were visualized under a fluorescence microscope.

### Cell migration and invasion

The cells were cultured in serum-free medium for 6 h, starved, digested with trypsin, and prepared to single cell suspension with serum-free medium. The cell concentration was adjusted to 5 × 10^5^ cells/mL, and 200 μL cell suspension was inoculated in the upper chamber (for the invasion assay, chambers were coated with Matrigel). 600 μL medium containing 5% FBS was set in the lower chamber. After 24 h of culture, the Transwell chamber was taken out, the medium in the chamber was sucked out, and the residual cells in the upper chamber were gently wiped off with a cotton swab. The Transwell chamber was fixed with 4% paraformaldehyde for 30 min, stained with 0.1% crystal violet dyeing solution for 1 h, and observed and photographed under inverted fluorescence microscope.

### RNA pulldown

RNA pulldown assay was performed according to the instructions of the RNA Pull-Down Kit (Thermo Fisher Scientific, Inc.). Briefly, full length of XIST sense/antisense was transcribed in vitro using Large Scale RNA Production Systems (Promega, USA) and labeled with Biotin using Biotin RNA Labeling Mix (Roche, Switzerland). Then 1 mg cell lysates extracted from osteosarcoma cells was incubated with 3 μg purified biotinylated transcripts for 1 h at 4 °C with rotation. Then the streptavidin agarose beads were added into cell protein lysate to precipitate the RNA-RNA complex. Elution buffers were used to elute the complex. The elute was collected, RNA was extracted with Trizol and stored at − 80 °C, and the amount of enriched miRNA was detected.

### Dual luciferase reporter assay

The psiCHECK-2 plasmid containing wild-type XIST(XIST^WT^) or mutated at the putative miR-655 binding sites (XIST^MT^) were designed, meanwhile the psiCHECK-2 plasmid containing wild-type ACLY 3’-UTR (ACLY 3’-UTR^WT^) or mutated at the putative miR-655 binding sites (ACLY 3’-UTR^MT^) were designed. When the cells reach 70% confluence, Lipofectamine™ 3000 was used to transfect cells with 2 μg plasmid and miR-655 agomir or NC. Dual luciferase Reporter Assay System (Promega, E1910) was used to detect the activity of firefly luciferase and Renillia luciferase: Add lysis buffer to lysed cells, Luciferase Assay Reagent II or Stop&Glo buffer were successively added,and detected by multi-functional enzyme marker.

### Prediction of the target genes of miR-655

The online database Starbase 3.0 (https://starbase.sysu.edu.cn) was used to predict the target genes of miR-655. In brief, the following steps were followed: (1) select miRNA-mRNA from miRNA-Target; (2) select miR-655-3p from microRNA; (3) select high stringency (≥ 3) from CLIP Data, select medium stringency (≥2) from Degrdome Data, select 4 programs from Program Number; (4) the obtained top three potential target genes ARPP19, TOB1, and ACLY;(5) then confirm miR-655 target genes through Western blot and dual luciferase reporter assay.

### Triglyceride and total cholesterol

RIPA lysate lysed cells or tissue samples for 40 min. Next, the cell homogenate was prepared for lipids extraction using chloroform/methanol (2:1). Intracellular triglyceride and total cholesterol were measured by using the triglyceride assay kit (Biovision, USA) and cholesterol assay kit (Biovision, USA) according to the manufacturers’ protocol, respectively.

### Animal model construction and treatment

The animal experiments were approved by the animal research committee of the Hunan Children's Hospital and were performed in accordance with established guidelines (approval No.: HCHDWLL-2022-01). Female BALB/c nude mice aged 4–6 weeks (purchased from Changzhou Cavens Experimental Animal Co., Ltd.) were fed under standard experimental animal feeding conditions: 21 °C, 12 h light–dark-cycle, with sufficient water and food supply. 143B cells stably expressing luciferase (143B/LUC) cells in logarithmic growth phase was used and the cell concentration was adjusted to 2 × 10^7^/mL, and 100 μL cell solution was injected into the tibial bone marrow cavity of BALB/c nude mice to establish an in-situ osteosarcoma model. One week after the cell injection, the mice were randomly divided into five group (n = 5): Ctrl, Exo^siNC^, Exo^siXIST^, Exo^siXIST^ + Anti-NC, Exo^siXIST^ + Anti-miR-655. The amount of exosome injection was 1 mg/kg, once every 3 days, a total of 6 times; the dosage of anti-NC or anti-miR-655 was 80 mg/kg, which was injected continuously for 3 days. The mice were anesthetized with isoflurane on the 0d, 14d and 28d after the cell inoculation. 150 mg/kg d-fluorescein was injected intraperitoneally, after 15 min later, the tumor growth and lung metastasis were detected by IVIS Lumina Imaging System (Xenogen). The mice were subsequently sacrificed, and the posterior limbs with tumors and the lungs were finely excised for further study. The tumor tissues were weighed, fixed for immunohistochemical and hematoxylin–eosin staining analysis, and lysed for western blotting and the numbers of metastatic lung nodules were counted using a dissecting microscope.

### Immunohistochemistry

The tumor tissue was fixed with 4% paraformaldehyde for 24 h, embedded in paraffin, and sliced into 4 μm slices, dewaxed in xylene, rehydrated with graded alcohol, and incubated with 3% H_2_O_2_ to block endogenous peroxidase activity. 10 mM sodium citrate (pH6.0) was boiled for 30 min for antigen repair, 5% BSA was used to block the slice for 30 min, and Anti-Ki67 antibody (Abcam, ab15580, 1:200) was added to incubate overnight at 4 °C. PBS was used to wash the slice for 3 times and the slice was incubated with secondary antibody at room temperature for 30 min. Images were captured using a microscope (Leica).

### Hemetoxylin-eosin staining

The lung tissue was fixed in 4% paraformaldehyde for 24 h and embedded in paraffin and 4 μm slices were prepared. Next, the sections were stained with hematoxylin solution for 5 min, incubated in 5% acetic acid, and then rinsed with tap water. Next, the sections were stained with eosin solution for 3 min and followed by dehydration with graded alcohol and clearing in xylene. At last, the sections were sealed, and the morphology of lung tissue was observed under a microscope.

### Statistical analysis

All statistical analysis were performed using graphpad prism 9.0. The quantitative data were shown as mean ± standard deviation (SD). The significance between the two groups were analyzed by Student's t-test. The significance between multiple groups were determined by one-way variance (ANOVA) analysis and LSD post hoc multiple comparison test. Statistical significance is indicated by *p < 0.05 and **p < 0.01.

## Results

### BMSCs derived exosomes increase the level of XIST in osteosarcoma cells

The cultured BMSCs were observed under light microscope, and the cells were spindle-shaped and wall-attached (Additional file 1: Fig. S1A). Then, MSC-specific surface markers (CD29 and CD90) and a hematopoietic marker CD45 were determined using flow cytometry, and the results showed that the positive rates of CD29 and CD90 were 99.72% ± 0.052% and 99.76% ± 0.035%, respectively, while the positive rates of CD45 was only 0.09% ± 0.017% (Additional file 1: Fig. S1B, C). Subsequently, ALP level analysis, Alizarin Red standing and Oil Red O standing confirmed that the cells had osteogenic and adipogenic differentiation potential (Additional file 1: Fig. S1D–F).

**Fig. 1:**
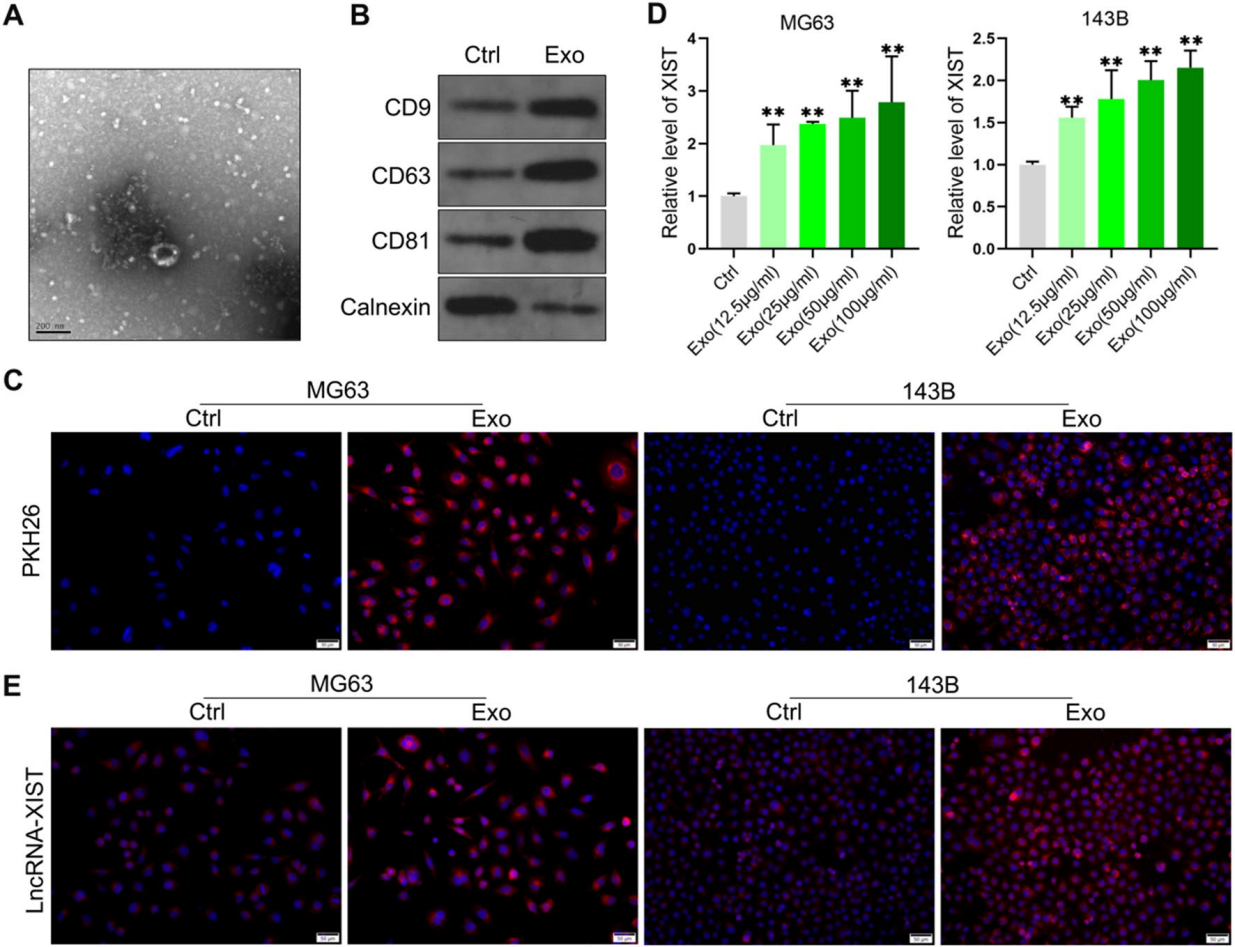
BMSCs derived exosomes increased the level of XIST in osteosarcoma cells. **A** The exosomes from BMSCs analyzed by TEM; **B** The levels of exosome markers CD9, CD63, CD81 and endoplasmic reticulum marker calnexin detected by western blot (n = 3); **C** PKH26-labeled exosomes internalized by osteosarcoma cellline MG63 and 143B; **D** qRT-PCR analysis of the level of XIST in osteosarcoma cell lines MG63 and 143B treated with different concentrations of exosomes from BMSCs (n = 3); **E** FISH results to observe the level and localization of XIST in osteosarcoma cell lines MG63 and 143B after treatment with BMSCs derived exosomes. ** represents p < 0.01

The exosomes from BMSCs were further isolated. Transmission electron microscopy (TEM) analysis showed that the exosomes from BMSCs were round membrane-bound vesicles with a size ranging from 40 to 100 nm in diameter (Fig. 1A). Western blot analysis showed that the exosome marker molecules CD9, CD63 and CD81 were highly expressed, and the endoplasmic reticulum marker molecule Calnexin was lowly expressed (Fig. 1B). BMSCs derived exosomes were labeled with PKH26 and then incubated with osteosarcoma cell lines MG63 and 143B, respectively. It was found that osteosarcoma cell lines MG63 and 143B could ingest BMSCs derived exosomes (Fig. 1C). Osteosarcoma cell lines MG63 and 143B were treated with BMSCs derived exosomes of different concentrations, and the level of XIST in cells was significantly increased (Fig. 1D). Fluorescence in situ hybridization (FISH) experiment also confirmed that the treatment with BMSCs derived exosomes on osteosarcoma cell lines MG63 and 143B could increase the level of XIST in these cells, and XIST was mainly localized in the cytoplasm (Fig. 1E). This indicated that osteosarcoma cells could uptake BMSCs derived exosomes, resulting in an increase in the level of XIST in the cells.

### BMSCs derived exosomes promotes osteosarcoma cell proliferation, migration and invasion through XIST

**Fig. 2:**
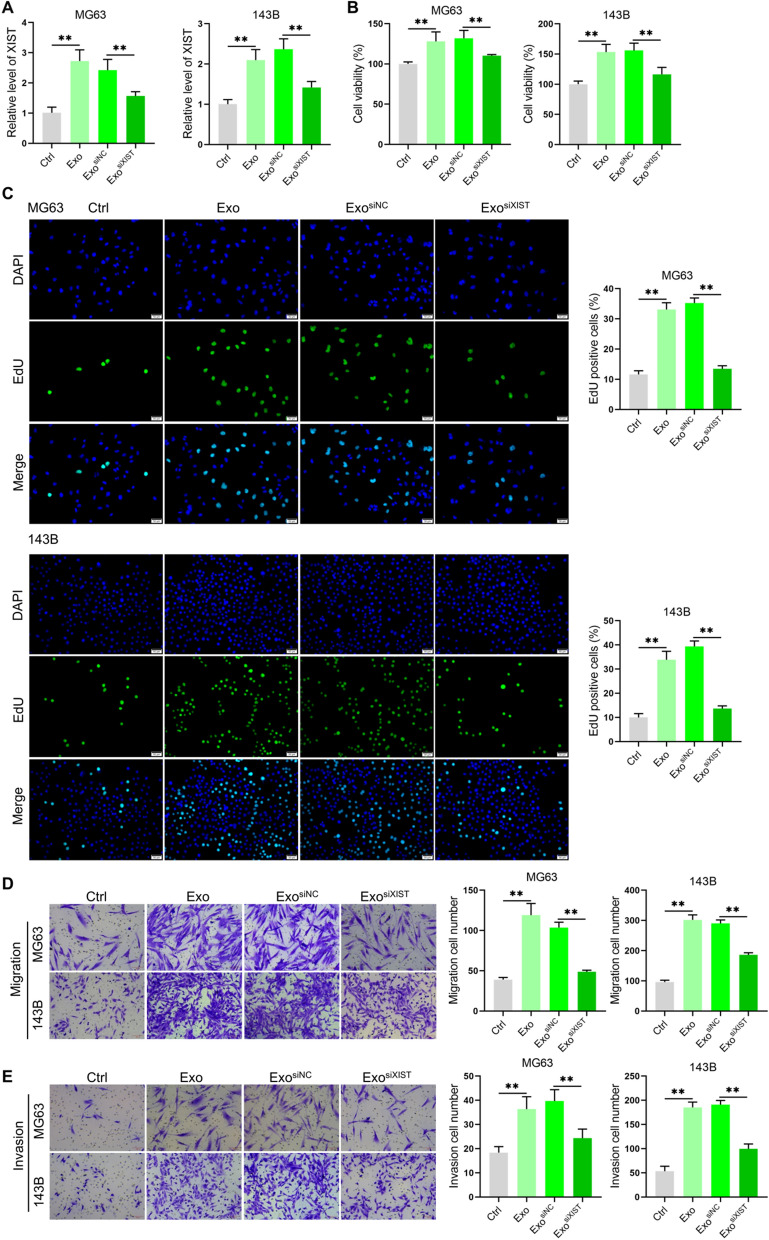
BMSCs derived exosomes promoted osteosarcoma cell proliferation, migration and invasion through XIST. A. The levels of XIST in osteosarcoma cell lines MG63 and 143B treated with exosomes analyzed by qRT-PCR(n = 3); **B** Cell viability of osteosarcoma cell lines MG63 and 143B after exosome treatment detected with CCK-8 kit(n = 3); **C** the proportion of EdU positive cells analyzed in osteosarcoma cell lines MG63 and 143B after they were treated with exosomes for 48 h, and 10 μM EdU was added and the cells were incubated for 4 h(n = 3); **D** Transwell chamber analysis for the migration ability of osteosarcoma cell lines MG63 and 143B after exosome treatment(n = 3); **E** Transwell Matrigel results to detect the changes of invasion ability of osteosarcoma cell lines MG63 and 143B after the exosome treatment(n = 3). **represents p < 0.01

Studies have shown that XIST can regulate the progression of osteosarcoma [23, 24], and BMSCs derived exosomes can increase the level of XIST in osteosarcoma cells. We speculated that BMSCs derived exosomes can affect osteosarcoma cell proliferation, migration and invasion through XIST. To verify this idea, XIST siRNA were synthesized and screened (Additional file 1: Fig. S2A). By extracting the exosomes derived from BMSCs after transfect siNC or siXIST, respectively, it was found that downregulated the level of XIST in BMSCs could significantly reduce XIST level in the exosomes (Additional file 1: Fig. S2B), after treating osteosarcoma cell lines MG63 and 143B, the level of XIST in osteosarcoma cells also decreased (Fig. 2A). Further functional analysis showed that BMSCs derived exosomes increased the osteosarcoma cell viability, DNA replication, migration and invasion, while down-regulating the level of XIST could significantly inhibit those effects (Fig. 2B–E). The results indicated that BMSCs derived exosomes could enhance osteosarcoma cell proliferation, migration and invasion through XIST.

### BMSCs derived exosomal XIST interact with miR-655 in osteosarcoma cells

**Fig. 3:**
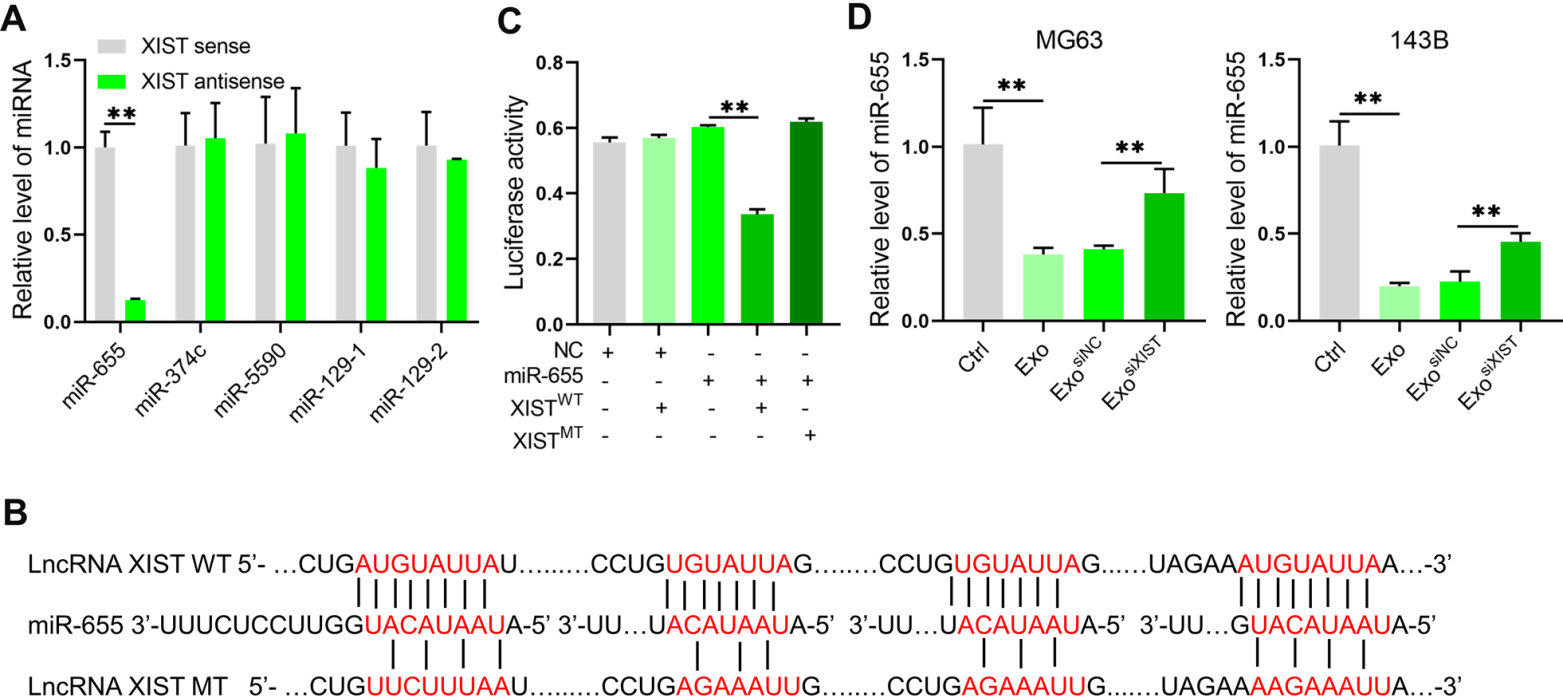
BMSCs derived exosomal XIST interacted with miR-655 in osteosarcoma cells. **A** RNA pulldown analysis to show that XIST bound miR-655 in osteosarcoma cell line MG63 (n = 3); **B** The binding sites and mutation information between XIST and miR-655; **C** Dual luciferase reporter gene assay results to show that XIST could bind miR-655 (n = 3); D. qRT-PCR results to confirm the effect of BMSCs derived exosomal XIST on the level of miR-655 in osteosarcoma cells MG63 and 143B (n = 3). **represents p < 0.01

The mechanism of lncRNA is closely related to its localization in cells. Based on previous studies, it was found that BMSCs derived exosomes XIST were mainly localized in the cytoplasm after entering osteosarcoma cells. We intended to explore the mechanism of BMSCs derived exosomal XIST promoting osteosarcoma progression through miRNA. With the online prediction of the miRNAs interacting with XIST through Starbase 3.0, we selected strict stringency (≥ 5) based on CLIP data and high stringency (≥ 3) based on Degradome data to obtain 12 miRNAs potentially binding to XIST. Then we analyzed the novelty and research status of those miRNAs in the field of cancer. We focused on miR-374c, miR-655, miR-5590, miR-129-1 and miR-129-2. In RNA pulldown experiment, it was found that XIST could bind to miR-655 (Fig. 3A). Further, we synthesized miR-655 agomir and antagomir, and verified their effects in osteosarcoma cell line MG63 (Additional file 1: Fig. S3). Dual luciferase reporter gene assay further confirmed the targeted relationship of XIST and miR-655 (Fig. 3B, C). Quantitative analysis showed that BMSCs derived exosomes could reduce the level of miR-655 in osteosarcoma cells. After inhibiting BMSCs XIST, the level of miR-655 in osteosarcoma cells was restored (Fig. 3D). These results indicated that the BMSCs derived exosomal XIST enter into osteosarcoma cells could bind to miR-655 and reduce the level of miR-655.

### BMSCs derived exosomal XIST promotes the osteosarcoma cell proliferation, migration and invasion by binding miR-655

**Fig. 4:**
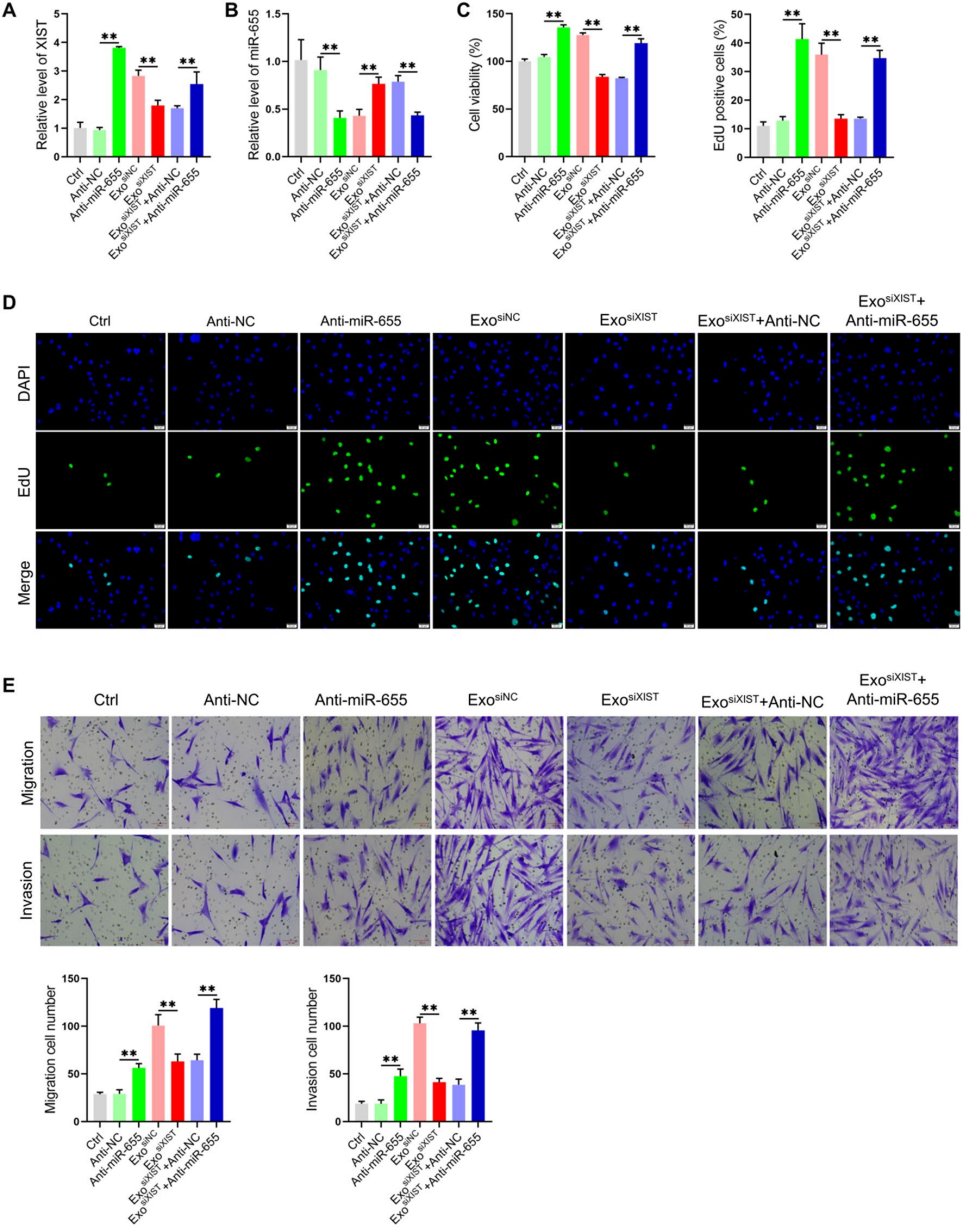
BMSCs derived exosomal XIST promoted osteosarcoma cell proliferation, migration and invasion by binding miR-655. qRT-PCR analyze the levels of XIST(**A**) and miR-655 (**B**) in osteosarcoma cells(n = 3); **C** CCK-8 assay results to show the viability of osteosarcoma cells(n = 3); **D** EdU labeling results to show the level of DNA replication(n = 3); **E** Transwell chamber results to detect cell migration and invasion(n = 3). **represents p < 0.01

Previous results showed that BMSCs derived exosomes XIST can bind and down-regulate the level of miR-655 in osteosarcoma cells. It is interesting to investigate whether miR-655 regulate the level of XIST. We found that inhibition of miR-655 could increase the level of XIST. In addition, inhibition of miR-655 could effectively antagonize the effect of BMSCs derived exosomes XIST on the level of XIST in osteosarcoma cells (Fig. 4A, B), suggesting that XIST and miR-655 in osteosarcoma cells could bind and regulate the level of each other, that is, there was a ceRNA mechanism. Studies have shown that miR-655 plays a role in inhibiting tumor progression in a variety of tumors, including hepatocellular carcinoma, esophageal squamous cell carcinoma and prostate cancer [25–27]. The decrease of the level of miR-655 In osteosarcoma tissues, the miR-655 level decreased, and inhibits osteosarcoma progression [28]. So the next question was whether the BMSCs derived exosomal XIST affect the biological function of osteosarcoma cells by binding miR-655. We found that inhibiting the expression of miR-655 increased the cell viability, the positive rates of EdU cells, the number of migrating and invasive cells, which again showed that miR-655 played a role in inhibiting osteosarcoma progression. On the basis of inhibiting XIST, downregulated miR-655 level restored the cell viability, DNA replication level, migration and invasion ability (Fig. 4C–E). These results suggested that BMSCs derived exosomal XIST can promote the osteosarcoma cell proliferation, migration and invasion by binding miR-655.

### BMSCs derived exosomal XIST promotes osteosarcoma progression by binding miR-655

**Fig. 5:**
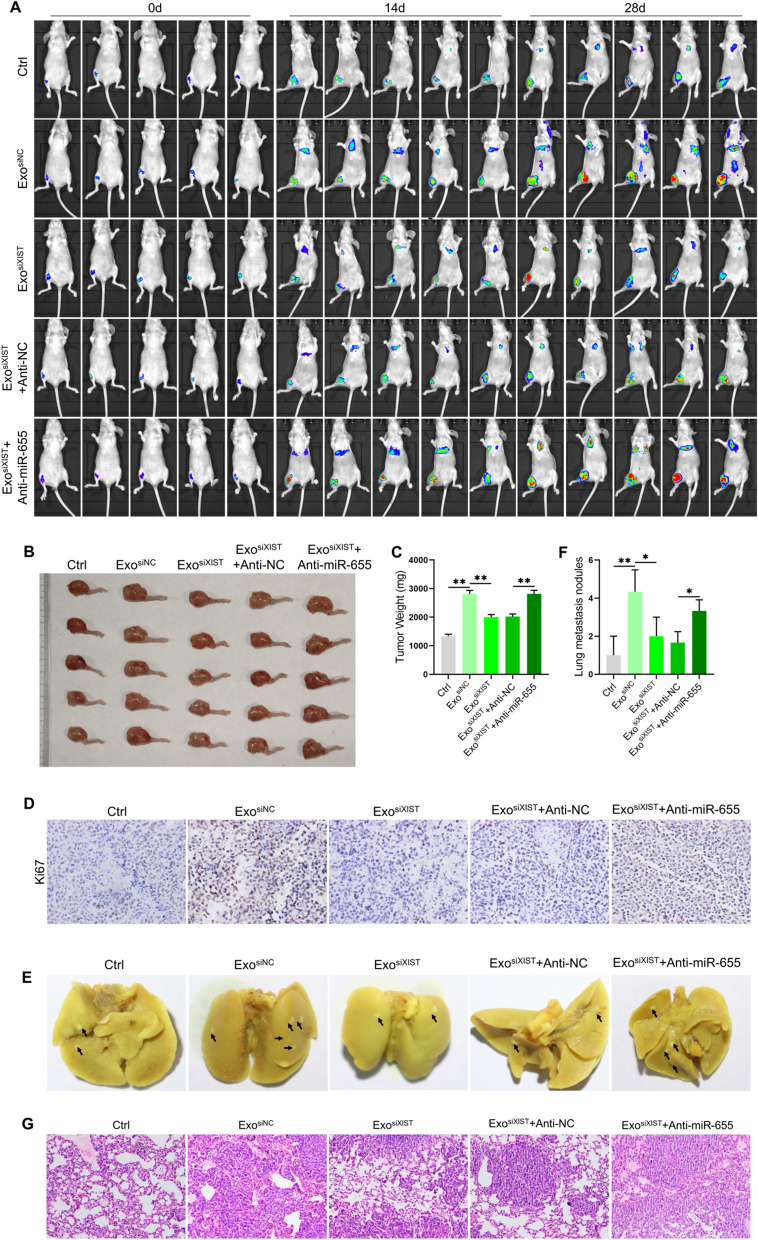
BMSCs derived exosomal XIST promoted osteosarcoma progression by binding miR-655. An in-situ osteosarcoma model was established by injecting 143B/LUC cells into the tibial bone marrow cavity of BALB/c nude mice. **A** The size and distribution of tumors in different treatment groups observed by in vivo imaging at 0, 14 and 28d respectively(n = 5); **B** The tumor size observed at 28d (n = 5); C. The weight of tumor tissues was measured (n = 5); **D** The positive staining of Ki67 in tumor tissue by immunohistochemical staining(n = 3); **E** The tumor metastasis observed after the lung tissue was dissected (n = 3); F. statistically analysis of the number of pulmonary metastases of osteosarcoma in different treatment groups(n = 3); **G** HE staining results to show the pathological changes of lung tissue(n = 3). *represents p < 0.05, ** represents p < 0.01

In order to further clarify the effect of BMSCs derived exosomal XIST combined with miR-655 on osteosarcoma, we established an osteosarcoma in situ model by injecting 143B/LUC cells into the tibial bone marrow cavity of BALB/c nude mice. The results showed that with the passage of time, the tumor volume and weight increased significantly, and obvious lung metastasis occurred at 28 days, while only a small number of lung metastases occurred at 14 days, and the metastatic tumors were small. Treated with BMSCs derived exosomes increased the tumor volume and weight, Ki67 positive staining was also significantly enhanced, the tumor metastasis was accelerated, and the number of lung metastases was significantly increased. However, down-regulation of XIST reduced tumor volume and weight, decreased Ki67 positive staining intensity, and inhibited lung metastasis. On this basis, downregulated miR-655 level, tumor volume and weight were significantly restored, Ki67 positive staining intensity was increased, and lung metastasis was also restored (Additional file 1: Fig.S4, Fig. 5A–G). These in vivo experimental results confirmed that BMSCs derived exosomes XIST can promote tumor growth and metastasis by binding miR-655.

### BMSCs derived exosomal XIST regulates ACLY expression in osteosarcoma cells by binding miR-655

**Fig. 6:**
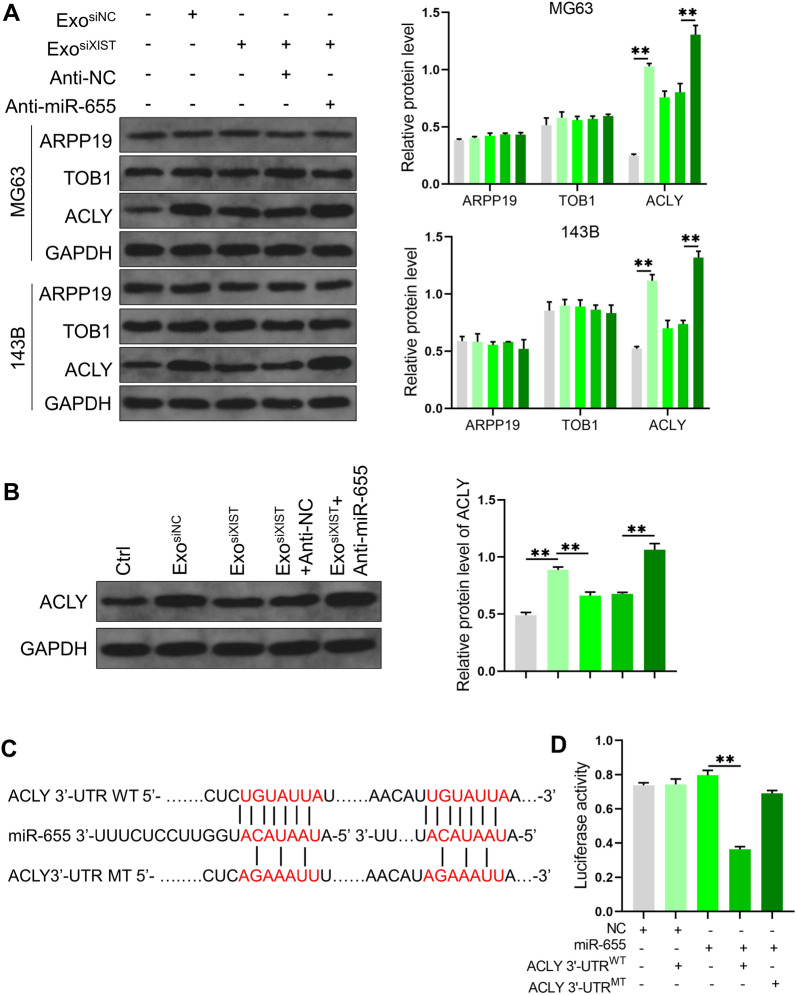
BMSCs derived exosomal XIST regulated ACLY expression in osteosarcoma cells by binding miR-655. **A** Western blot analysis the effect of BMSCs derived exosomal XIST combined with miR-655 on the expression of ARPP19, TOB1 and ACLY in osteosarcoma cells (n = 3); **B** At animal level, western blot analysis the effect of BMSCs derived exosomal XIST combined with miR-655 on ACLY expression (n = 3); **C** The binding pattern of miR-655 and the ACLY 3'-UTR and the corresponding mutation information; **D** The binding of miR-655 and the ACLY 3'-UTR and their possible sites analyzed by dual luciferase reporter gene assay (n = 3). ** represents p < 0.01

In order to further clarify the mechanism of promoting osteosarcoma progression by BMSCs derived exosomal XIST combined with miR-655, we first analyzed the target gene of miR-655 through Starbase 3.0. We set clip data as high stringency (≥3), Degrdome data as medium stringency (≥2), and Program number as 4 programs. The top 3 genes were ARPP19, TOB1, and ACLY, respectively. It was found that BMSCs derived exosomes could promote the expression of ACLY in osteosarcoma cells, inhibition of XIST expression reduces ACLY level. On this basis, downregulated miR-655 level, the expression of ACLY was restored. However, the protein levels of ARPP19 and TOB1 did not change among the treatment groups (Fig. 6A). At the in vivo level, we also found a similar phenomenon (Fig. 6B). Further, we confirmed by dual luciferase reporter gene assay that miR-655 bound to the ACLY 3 '-UTR. After mutated at the putative miR-655 binding sites, the relative luciferase activity was restored (Fig. 6C, D), which indicated that BMSCs derived exosome XIST can promote the expression of ACLY in osteosarcoma cells by binding to miR-655.

### BMSCs derived exosomal XIST promotes ACLY expression by binding miR-655, thereby enhancing lipid synthesis and β-catenin signal activity

**Fig. 7:**
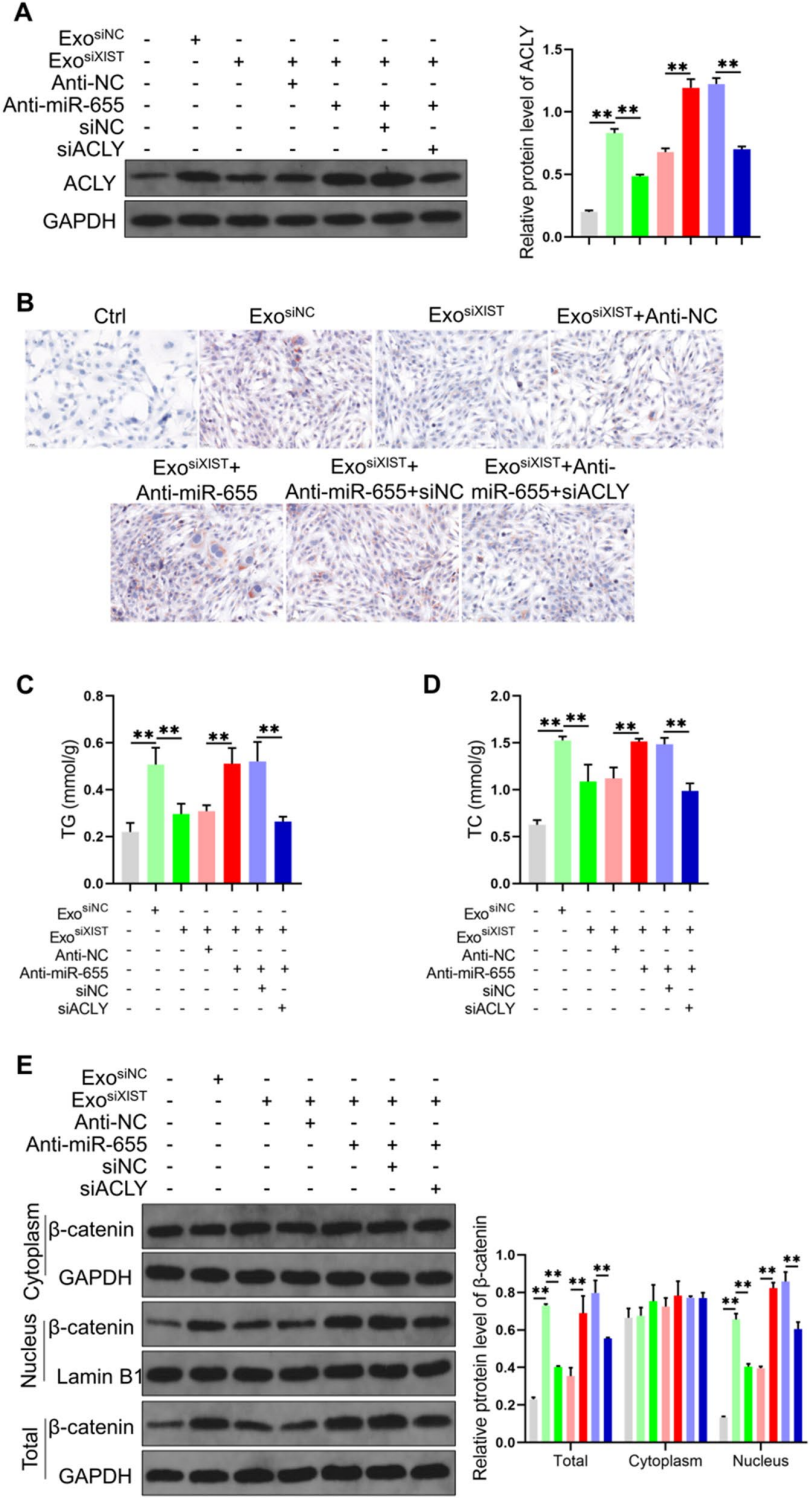
BMSCs derived exosomal XIST promoted ACLY expression by binding miR-655, thereby enhancing lipid synthesis and β-Catenin signal activity. **A** Quantitative detection and analysis of ACLY level in cells (n = 3); **B** Lipid deposition in cells analyzed by Oil Red O staining(n = 3); the levels of TG (**C**) and TC (**D**) in the cells detected by the kit (n = 3); **E** Western blot analysis of the content of β-catenin in cell total, cytoplasmic and nuclear protein(n = 3). ** represents p < 0.01

Prior studies have shown that ACLY is promotes lipid synthesis and enhancing β-catenin signaling [29, 30], while the elevation of lipid synthesis and β-catenin signaling activity can accelerate tumor progression [31, 32]. While BMSCs derived exosomal XIST was found able to promote ACLY expression by binding miR-655, it was interesting to study how the above effect promotes lipid synthesis and enhances β-catenin signal activity, which is currently unknown. We found that BMSCs derived exosomes promoted lipid accumulation in osteosarcoma cells, significantly increased triglyceride (TG) and total cholesterol (TC) levels, decreased lipid deposition levels in cells after inhibiting XIST, on this basis, downregulated miR-655 level, the lipid levels, TG and TC contents were also restored, Inhibit the expression of ACLY, the level of TG and TC decreased accordingly (Fig. 7A–D). Analysis of β-catenin expression and its protein content in cytoplasm and nucleus, we found that BMSCs derived exosomes increased the protein level of β-catenin in total and nuclear protein, after inhibiting XIST, the content of β-catenin in total and nuclear protein decreased, on this basis, after the expression of miR-655 was down-regulated, the content of β-catenin in total and nuclear protein restored; after the expression of ACLY was inhibited, the content of β-catenin in total protein and nuclear protein decreased accordingly (Fig. 7E). These results suggested that BMSCs derived exosomal XIST can promote ACLY expression by binding miR-655, thus promoting lipid accumulation and increasing β-catenin on total and on the nuclear level.

**Fig. 8:**
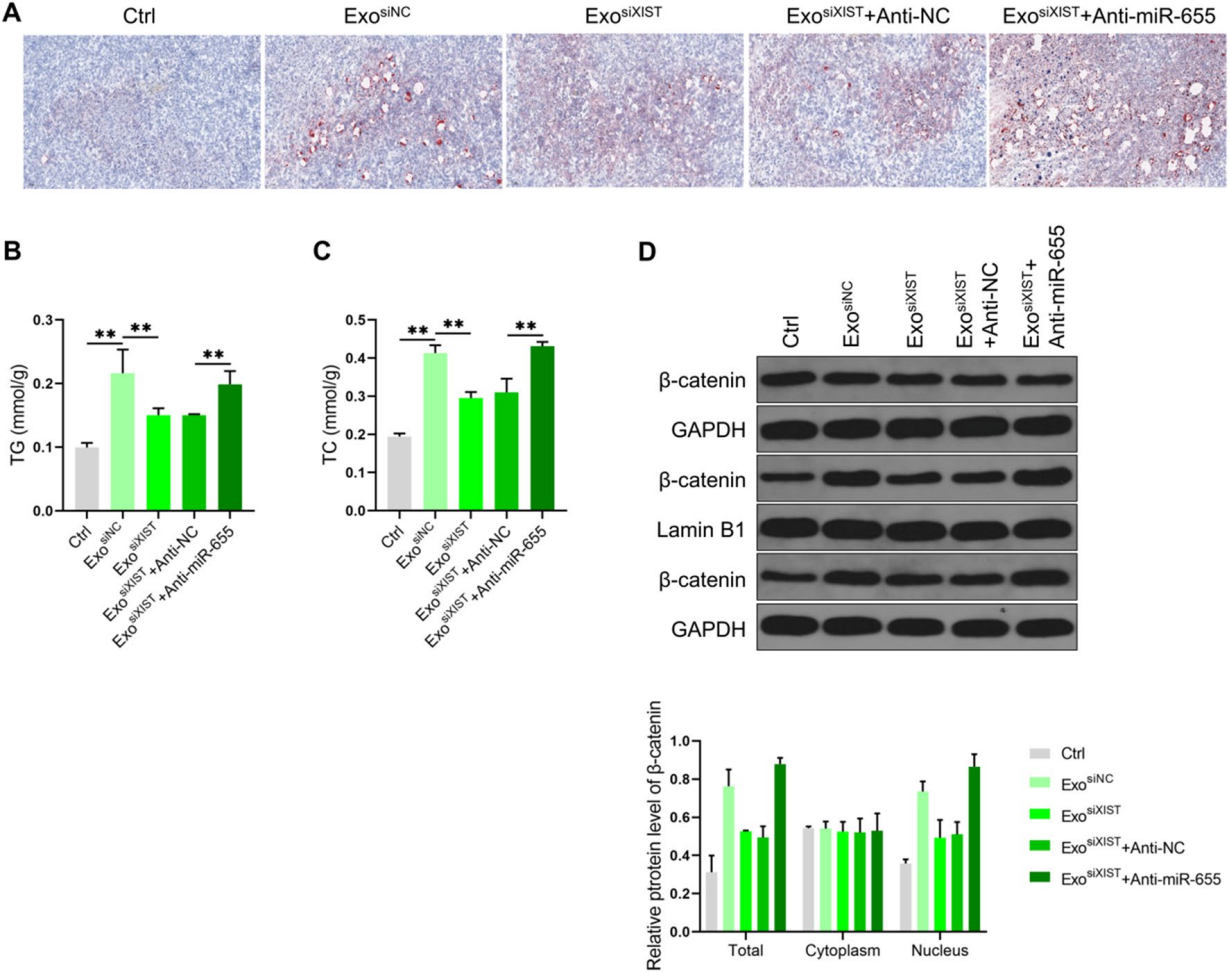
Animal level validation of the effect of BMSCs derived exosomal XIST on lipid synthesis and the activity of β-catenin. **A** The level of lipid deposition in osteosarcoma tissue samples analyzed by Oil Red O staining (n = 3); the levels of TG **B** and TC **C** in osteosarcoma tissue samples detected with the kit (n = 3); D. Western blot analysis of the content of β-catenin in total, cytoplasmic and nuclear protein in osteosarcoma tissue samples(n = 3). ** represents p < 0.01

We further verified the results at the animal level. It was found that BMSCs derived exosomes promoted lipid deposition and increased TG and TC levels in osteosarcoma tissues. After inhibiting XIST, lipid deposition and TG and TC levels decreased. On this basis, after downregulated miR-655 level, lipid deposition, TG and TC levels were restored (Fig. 8A–C). We also analyzed the level of β-catenin, and it was found that the exosomes from BMSCs increased the content of β-catenin in total and nuclear protein, after inhibiting XIST, the content of β-catenin in total and nuclear protein decreased. On this basis, after downregulated miR-655 level, the content of β-catenin in total and nuclear protein levels was restored (Fig. 8D). These results were consistent with the cell level. It was confirmed that BMSCs derived exosomal XIST can promote ACLY expression by binding miR-655, thus promoting lipid accumulation and enhancing β-catenin signal activity.

### BMSCs derived exosomal XIST enhances ACLY expression by binding miR-655, thereby promoting the osteosarcoma cell proliferation, migration and invasion

**Fig. 9:**
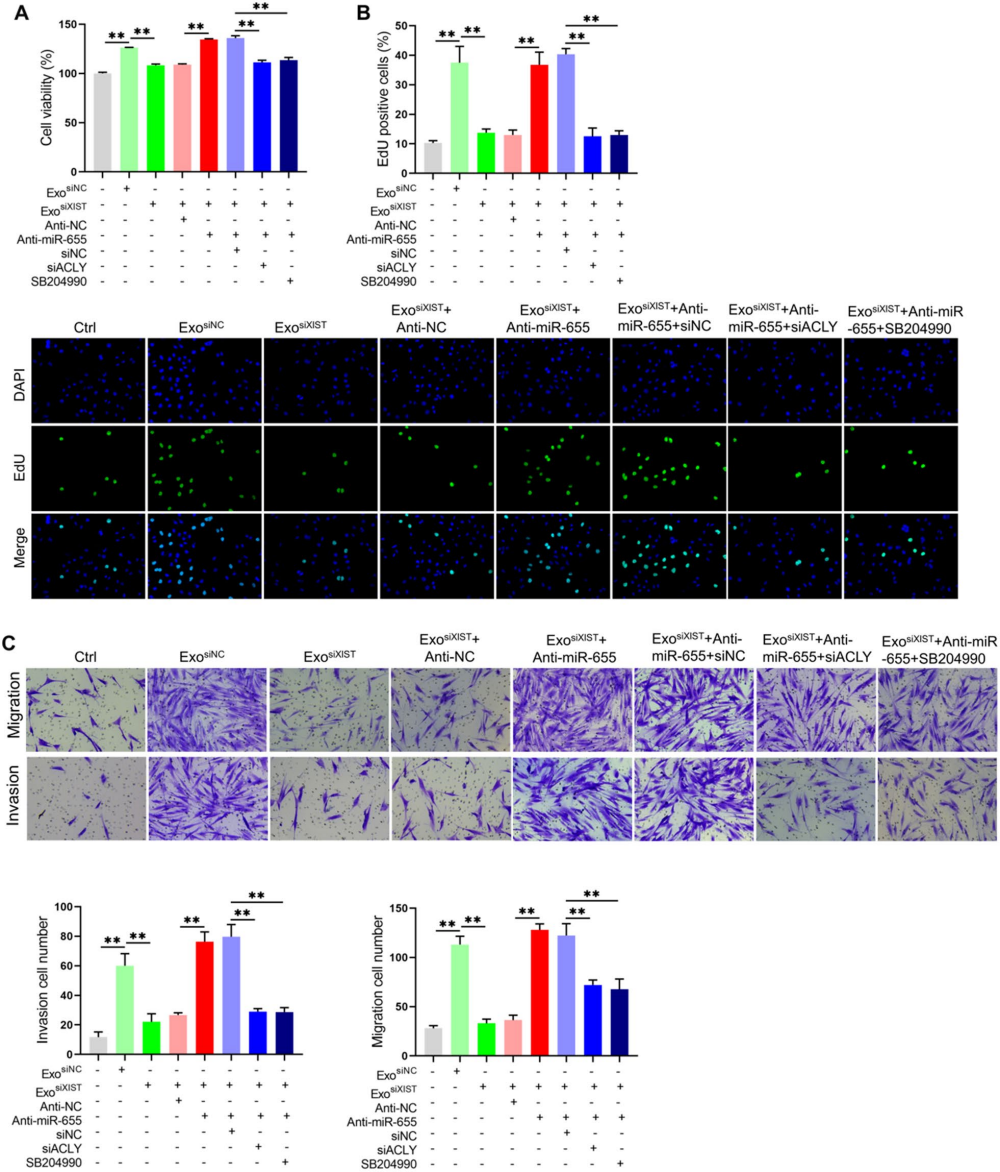
BMSCs derived exosomal XIST enhanced ACLY expression by binding miR-655, thereby promoting the proliferation, migration and invasion of osteosarcoma cells. **A** CCK-8 kit analysis for cell viability (n = 3); **B** The proportion of EdU positive cells after 10 μM EdU was added and incubated for 4 h (n = 3); **C** Transwell chamber analysis to show the migration and invasion of cells in each group (n = 3). ** represents p < 0.01

Finally, we analyzed the effect of BMSCs derived exosomal XIST on the biological function of osteosarcoma by enhancing ACLY expression in combination with miR-655. The results showed that BMSCs derived exosomal XIST increased the cell viability and increased the proportion of EdU positive cells by binding miR-655. On this basis, inhibition of ACLY expression or treatment with ACLY inhibitor SB204990 decreased the cell viability and the proportion of EdU positive cells (Fig. 9A, B). There results showed that BMSCs derived exosomal XIST could increase the ACLY level by binding miR-655, thereby promoting the proliferation of osteosarcoma cells. Similarly, through migration and invasion assays, it was found that BMSCs derived exosomal XIST could promote the migration and invasion by combining miR-655, on this basis, inhibiting the expression of ACLY or treating with SB204990 would reduce the migration and invasion ability of osteosarcoma cells (Fig. 9C). These results indicated that BMSCs derived exosomal XIST can increase ACLY expression by binding to miR-655, thereby enhancing the migration and invasion of osteosarcoma.

## Discussion

Osteosarcoma mostly occurs in the epiphysis of long bone marrow, such as femur, tibia and humerus. It is characterized by the formation of immature bone or osteoid tissue by tumor cells [33]. BMSCs derived exosomes can promote the malignant progression of osteosarcoma, but the mechanism is still insufficient, especially the role and mechanism based on lncRNA still need to be revealed. In this study, we found that BMSCs derived exosomes can deliver XIST into osteosarcoma cells, bind and down-regulate the level of miR-655 through ceRNA mechanism, thus leading to increase of ACLY expression, and promoting lipid synthesis, enhanced β-catenin signal activity, accelerates the growth and metastasis of osteosarcoma.

As the progenitor cells derived from bone marrow, BMSCs have the potential of self-renewal and multi differentiation [34]. In different tumors, BMSCs play different roles. For example, BMSCs promote the proliferation and angiogenesis of breast cancer and prostate cancer [35], but inhibit the progression of glioma and hepatocellular carcinoma, this effect should be related to their derived exosomes [36, 37]. There are similar phenomena in osteosarcoma. Most studies have shown that BMSCs promote the progression of osteosarcoma, and its mechanism includes the role of transferring exosome [38]. Interestingly, Zhou et al. found that BMSCs can transfer miR-1913 into osteosarcoma cells through exosomes, reduce the expression level of NRSN2, and play a role in inhibiting the proliferation, migration and invasion of osteosarcoma [39]. Our study confirmed that BMSCs derived exosomes can promote the osteosarcoma cell proliferation, migration and invasion in vitro and the growth and lung metastasis in vivo, which is consistent with the conclusions of most studies.

XIST is the first found lncRNA to play a key role in X chromosome inactivation [40]. Subsequent studies have shown that XIST is involved in tumor progression. However, whether it is an oncogene or a tumor suppressor gene is not clear, and both sides has supporting reports. Even in the same tumor, there are inconsistent research results, including hepatocellular carcinoma, breast cancer, ovarian cancer and renal cell carcinoma [15]. This phenomenon also exists for osteosarcoma. Most studies support that XIST plays a role as an oncogene. In addition to the mechanisms described above, it also includes promoting RSF1 expression by combining miR-193a-3p, promoting RAP2B expression by combining miR-320b, and recruiting methyltransferase EZH2 to promote the modification of P21 promoter H3K27m3, thereby inhibiting P21 expression [23, 24, 41]. However, Zhang et al. found that the level of XIST in osteosarcoma decreased, and its level is negatively correlated with the overall survival of patients, its anti-cancer effect is related to the combination of miR-21-5p and then promotes PDCD4 expression [42]. We found for the first time that BMSCs derived exosomes can transmit XIST to promote osteosarcoma progression. After down-regulating the expression of XIST, the cell proliferation, migration and invasion in vitro, tumor growth and lung metastasis in vivo were inhibited, indicating that BMSCs derived exosomal XIST play a role in promoting cancer, which is consistent with most research reports.

The mechanism of the function of XIST mainly includes binding chromatin modifying molecules and binding miRNAs as molecular sponges to regulate the expression of target molecules [15]. We found that XIST was mainly localized in the cytoplasm of osteosarcoma cells. When treated with BMSCs derived exosomes, the staining intensity of XIST in the cytoplasm was significantly increased. Ii is considered that the place where XIST binds to chromatin modifying molecules is in the nucleus, and the place where XIST acts as a molecular sponge to bind miRNA is in the cytoplasm, we intended to preliminarily explore the mechanism of the BMSCs derived exosomal XIST from the perspective of miRNA. The results showed that BMSCs derived exosomal XIST could bind miR-655 based on ceRNA mechanism after it enter into osteosarcoma cells. miR-655 is considered as a regulatory molecule with therapeutic potential, and has attracted more and more attention in recent years [43]. The related research reports mainly focus on the field of tumor, as a tumor suppressor gene to regulate cell proliferation, apoptosis, migration, invasion, angiogenesis, EMT and other processes, and the tumor kinds includes oesophageal square cell carcinoma, prostate cancer and triple negative blast cancer [25, 26, 44]. In osteosarcoma, the level of miR-655 decreased, LINC00689 can increase the expression level of SOX18 by combining with miR-655, thus promoting the osteosarcoma cell proliferation, migration and invasion [28]. It is preliminarily suggested that miR-655 plays a role in inhibiting the progression of osteosarcoma. In this study, we found that inhibiting the expression of miR-655 alone significantly enhanced the osteosarcoma cell proliferation, migration and invasion, which once again confirmed the role of miR-655 in inhibiting osteosarcoma progression. On the basis of down-regulation of XIST, after the expression of miR-655 in osteosarcoma cells was inhibited, the cell proliferation, migration and invasion in vitro, tumor growth and metastasis in vivo were significantly restored. This indicated that BMSCs derived exosomal XIST can promote osteosarcoma progression by binding miR-655.

miRNA plays a role in a variety of mechanisms [45], of which the most widely studied one is to regulate the gene expression by targeting and binding the 3'-UTR of the target molecule. Through bioinformatics and experimental studies, we confirmed that ATP citrate lyase (ACLY) is a target gene of miR-655. ACLY plays a role in promoting cancer in a variety of tumors, including osteosarcoma [45], and targeting ACLY is considered to be one of the important strategies for tumor prevention and treatment [46]. We found that BMSCs derived exosomal XIST combined with miR-655 could increase the expression level of ACLY and promote the proliferation, migration and invasion of osteosarcoma cells.

At present, studies have shown that ACLY can catalyze citric acid to produce Acetyl CoA (AcCoA) in the cytoplasm, which is the raw material for the synthesis of fatty acids and mevalonate, further to generate lipids. Therefore, it is widely reported that ACLY can promote lipid synthesis [30]. There are many kinds of lipids in organisms, their functions include energy storage, oxidation and energy supply, forming lipid bilayer of biofilm, and participating in cell recognition and signal transmission. The rapid proliferation and metastasis of tumor cells require a lot of material and energy, and lipids can support this demand [32, 47]. In this study, we failed to fully reveal the effect of BMSCs exosomal XIST on the lipid expression profile in osteosarcoma cells by mass spectrometry, but by analyzing the common and key lipid levels of TG and TC, we found that BMSCs derived exosomes increased the TG and TC levels in cells and tissues. After inhibiting XIST, the TG and TC levels decreased, and this effect was related to the combination of miR-655 and the promotion of ACLY expression. Similarly, we also found a similar trend through Oil Red O staining experiment, which preliminarily suggests that BMSCs derived exosomal XIST combined with miR-655 promotes ACLY expression, and then regulates the lipid level in cells, which is one of the mechanisms of accelerating the growth and metastasis of osteosarcoma.

In addition, ACLY can be combined with β-catenin and enhance its stability, promote its transfer from cytoplasm to nucleus, and enhance its transcriptional regulation activity, while β-catenin has been recognized to function as a promoting role in tumor progression, including regulating the expression of cyclin and EMT process related molecules, thus promoting the growth and metastasis of tumor cells [29]. At the same time, it is also reported that XIST can enhance the signaling activity of β-catenin, whose mechanism includes binding miR-1264 to promote WNT5A expression, binding miR-34a to promote WNT1 expression, binding miR-139 to promote WNT1 and β-catenin expression and binding miR-744/RING1 to enhance the signal activity of Wnt/β-catenin [48–51]. In this study, we confirmed that the BMSCs derived exosomal XISTcould increase the level of β-catenin and promote its entry into nucleus, and the promotion of ACLY expression by binding miR-655 is the mechanism of its effect. BMSCs derived exosomal XIST binds miR-655 to promote ACLY expression, and then enhances β-catenin signal activity, which is another mechanism of XIST to accelerate the growth and metastasis of osteosarcoma.

Therefore, this study confirmed that BMSCs derived exosomes can transfer XIST into osteosarcoma cells, promote the growth and metastasis of osteosarcoma, combine with miR-655 and down-regulate its level to promote ACLY expression, resulting in increased lipid synthesis and the increase of signal activity of β-catenin, which is the mechanism of the action of XIST.Although the collection of large clinical samples is a time-consuming and demanding work, the preclinical research results gave us confidence. We plan to collect peripheral blood serum samples of osteosarcoma patients at different stages, isolate and detect the XIST level in serum exosomes, so as to clarify the difference between osteosarcoma patients and normal people, as well as the difference between the XIST level in serum exosomes of osteosarcoma patients at different stages. We plan to analyze the value of BMSCs derived exosomal XIST as a disease biomarker and for postoperative monitoring, as well as preclinical research and clinical research results, which will provides a theoretical basis for the development of new treatment strategies, especially for osteosarcoma patients with lung metastatic and with poor prognosis.

## Supplementary Information

**Additional file 1: Figure S1.** Characterization of BMSCs. **A** Morphology of BMSCs under light microscope; **B**, **C** Flow cytometry and statistical analysis of the positive rates of MSC-specific surface markers (CD29 and CD90) and a hematopoietic marker CD45 (n = 3); **D** The analysis of ALP level after osteogenesis induction 14d(n = 3); **E** The level of mineralized nodules analyzed with Alizarin red staining after osteogenesis induction 21d (n = 3); **F** The level of lipid deposition analyzed with Oil Red O staining after lipogenesis induction (n = 3). **represents p < 0.01. **Figure S2.** XIST siRNA reduced XIST levels in BMSCs and their secreted exosomes. **A** qRT-PCR results to screen siRNA that specifically down regulated the level of XIST in BMSCs(n = 3); **B** qRT-PCR analysis of the effect of down-regulation of the level of XIST in BMSCs on the content of XIST in their secreted exosomes (n = 3). **represents p < 0.01. **Figure S3.** MiR-655 agomir and antagomir were transfected into MG63 cells, after 48 h, the miR-655 level was detected by qRT-PCR (n = 3). **represents p < 0.01. **Figure S4.** qRT-PCR analysis the levels of XIST and miR-655 in osteosarcoma tissue (n = 3). **represents p < 0.01. **Figure S5.** ACLY specific siRNA screening. SiNC and siACLY were transfected into MG63 cells, after 72 h, the ACLY level was detected by qRT-PCR(n = 3). **represents p < 0.01.

## Data Availability

The data that support the findings of this study are available from the corresponding author upon reasonable request.
