# Supplementary material for: LncRNA XIST from the bone marrow mesenchymal stem cell derived exosome promotes osteosarcoma growth and metastasis through miR-655/ACLY signal
Source: Cancer Cell Int. 2022 Oct 29;22:330. doi: 10.1186/s12935-022-02746-0 (PMC9617450; doi:10.1186/s12935-022-02746-0)
Supplement: Supplementary file 1 — Additional file 1: Figure S1. Characterization of BMSCs. A Morphology of BMSCs under light microscope; B, C Flow cytometry and statistical analysis of the positive rates of MSC-specific surface markers (CD29 and CD90) and a hematopoietic marker CD45 (n = 3); D The analysis of ALP level after osteogenesis induction 14d(n = 3); E The level of mineralized nodules analyzed with Alizarin red staining after osteogenesis induction 21d (n = 3); F The level of lipid deposition analyzed with Oil Red O staining after lipogenesis induction (n = 3). **represents p < 0.01. Figure S2. XIST siRNA reduced XIST levels in BMSCs and their secreted exosomes. A qRT-PCR results to screen siRNA that specifically down regulated the level of XIST in BMSCs(n = 3); B qRT-PCR analysis of the effect of down-regulation of the level of XIST in BMSCs on the content of XIST in their secreted exosomes (n = 3). **represents p < 0.01. Figure S3. MiR-655 agomir and antagomir were transfected into MG63 cells, after 48 h, the miR-655 level was detected by qRT-PCR (n = 3). **represents p < 0.01. Figure S4. qRT-PCR analysis the levels of XIST and miR-655 in osteosarcoma tissue (n = 3). **represents p < 0.01. Figure S5. ACLY specific siRNA screening. SiNC and siACLY were transfected into MG63 cells, after 72 h, the ACLY level was detected by qRT-PCR(n = 3). **represents p < 0.01. [file 12935_2022_2746_MOESM1_ESM.docx]

**Supplementary figures**

**
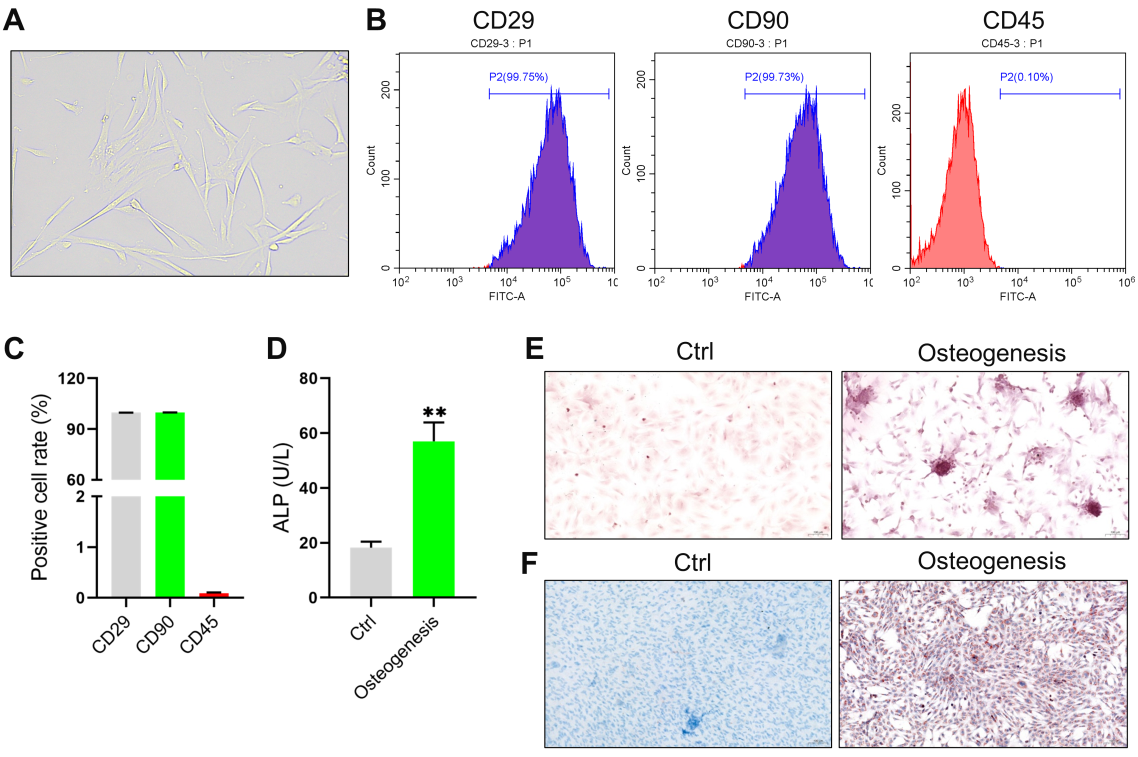
**

**Figure S1.** Characterization of BMSCs. A. Morphology of BMSCs under light microscope; B&C. Flow cytometry and statistical analysis of the positive rates of MSC-specific surface markers (CD29 and CD90) and a hematopoietic marker CD45(n=3); D. The analysis of ALP level after osteogenesis induction 14d(n=3); E. The level of mineralized nodules analyzed with Alizarin red staining after osteogenesis induction 21d(n=3); F. The level of lipid deposition analyzed with Oil Red O staining after lipogenesis induction(n=3). **represents p<0.01.


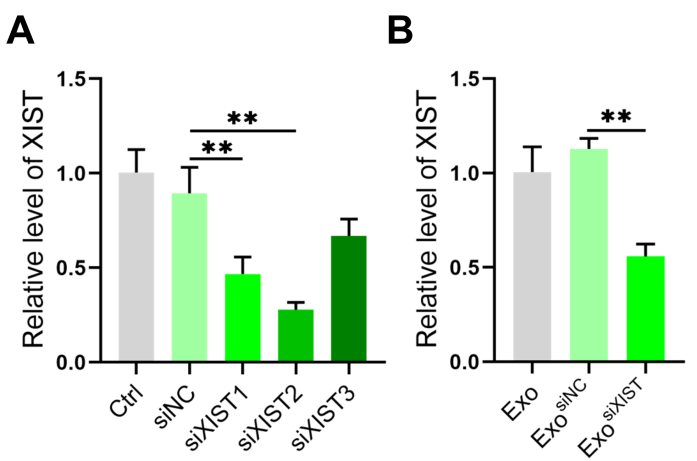


**Figure S2.** XIST siRNA reduced XIST levels in BMSCs and their secreted exosomes. A. qRT-PCR results to screen siRNA that specifically down regulated the level of XIST in BMSCs(n=3); B. qRT-PCR analysis of the effect of down-regulation of the level of XIST in BMSCs on the content of XIST in their secreted exosomes(n=3). ** represents p<0.01.

**
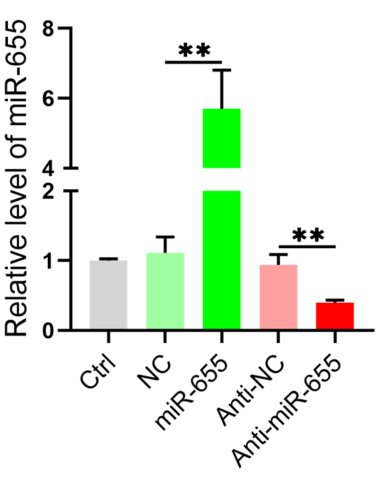
**

**Figure S3.** MiR-655 agomir and antagomir were transfected into MG63 cells, after 48h, the miR-655 level was detected by qRT-PCR(n=3). ** represents p<0.01.

**
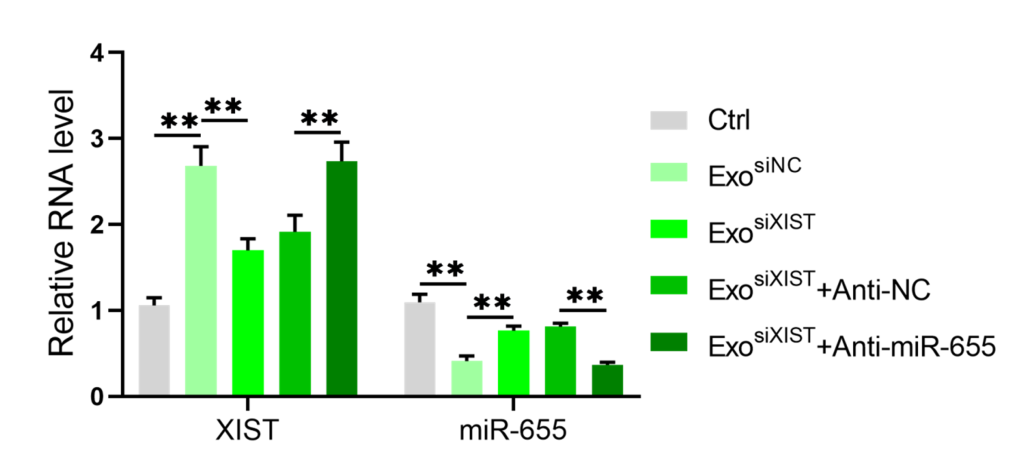
**

**Figure S4.**qRT-PCR analysis the levels of XIST and miR-655 in osteosarcoma tissue(n=3). ** represents p<0.01.

**
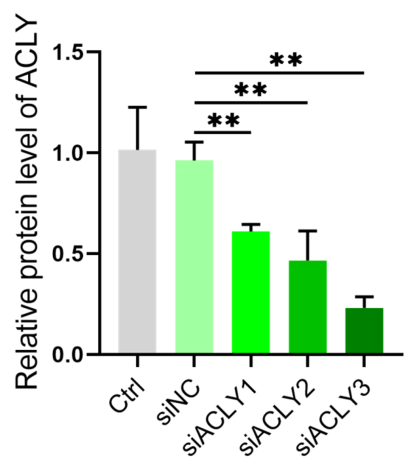
**

**Figure S5.** ACLY specific siRNA screening. SiNC and siACLY were transfected into MG63 cells, after 72h, the ACLY level was detected by qRT-PCR(n=3). ** represents p<0.01.
